# The Chronological Evolution of Cochlear Implant Contraindications: A Comprehensive Review

**DOI:** 10.3390/jcm13082337

**Published:** 2024-04-18

**Authors:** Nezar Hamed, Norah Alajmi, Faisal Ibrahim Alkoblan, Yazeed Abdullah Alghtani, Yassin Abdelsamad, Ahmed Alhussien, Rafeef Ibrahim Alhajress, Salman F. Alhabib

**Affiliations:** 1 King Abdullah Ear Specialist Center (KAESC), College of Medicine, King Saud University Medical City (KSUMC), King Saud University, P.O. Box 245, Riyadh 11411, Saudi Arabia; alajmin@outlook.sa (N.A.); faisal.alkoblan1@gmail.com (F.I.A.); alghtani.yazeed@gmail.com (Y.A.A.); alhussienah@gmail.com (A.A.); rafeefalhajress@gmail.com (R.I.A.); 2 Research Department, MED-EL GmbH, P.O. Box 245, Riyadh 11411, Saudi Arabia; yassin.samad@medel.com

**Keywords:** cochlear implant, contraindications, hearing loss, complications, outcomes

## Abstract

Cochlear implantation has emerged as a transformative intervention in addressing profound hearing loss, offering a paradigm shift in auditory rehabilitation for individuals with restricted auditory function. Throughout its history, the understanding of contraindications for cochlear implant (CI) surgery has evolved significantly. This review comprehensively analyzes the chronological advancements in the understanding of CI contraindications, examining studies conducted from historical timelines to the present. Recent research has revealed significant developments in the field, prompting a reevaluation of established criteria and resulting in expanded indications for CI. The chronological evolution of contraindications underscores the transformative nature of the field, offering potential improvements in outcomes and enhancing the quality of life for individuals with profound hearing loss. In conclusion, this narrative review emphasizes the dynamic nature of the field, where the reevaluation of contraindications has created new opportunities and broader indications for CI. The emerging prospects, including improved outcomes and enhanced quality of life, hold promise for individuals with profound hearing loss.

## 1. Introduction

The cochlear implant (CI) stands as a groundbreaking intervention in the field of otology, offering a remarkable solution to hearing loss, which is one of the most disabling disabilities worldwide [1]. Over the past few decades, notable advancements in CI devices, electrodes, and surgical techniques have ushered in a new era [2], broadening patient eligibility and extending the transformative impact of this intervention.

The landscape of CI contraindications has experienced significant evolution throughout its historical trajectory. CI contraindications are defined as medical or anatomical circumstances indicating that CI may not be suitable for an individual. Conditions once considered absolute contraindications, such as otitis media, single-sided deafness, residual hearing, cochlear ossification, cochlear fracture, retrocochlear pathology, Ménière’s disease, inner ear malformations, significant psychiatric disorders, cognitive impairment, age limitations, and medical comorbidities, have undergone rigorous reevaluation, emphasizing the dynamic nature of CI technology and outcomes understanding. CIs nowadays represent a viable option for individuals with varying degrees of hearing impairment, including those with residual hearing [2], challenging traditional contraindications and embracing personalized approaches to CI [3,4,5]. Advancements in technology and surgical techniques, including minimally invasive approaches, have not only mitigated risks associated with CI surgery but have also broadened the spectrum of eligible patients [4].

Audiological considerations have also played a crucial role in the reevaluation of contraindications. Comprehensive audiological assessments, including measures of residual hearing and auditory processing abilities [6,7], have provided valuable insights into the potential benefits of CIs in individuals previously considered unsuitable candidates. Moreover, psychosocial factors, such as communication needs and quality of life considerations, have gained recognition as essential components in the decision-making process.

The efficacy of CI outcomes is influenced by a myriad of factors, encompassing the underlying biology of the hearing loss and various post-implantation variables. Extensive research underscores the impact of factors such as the etiology of hearing loss, whether peripheral or central, along with genetic predispositions, which can significantly shape the success of CI interventions [8]. Moreover, the extent of receptor damage or neuronal impairment, stemming from diverse etiologies, may intricately affect the effectiveness of CIs [9]. Crucially, tailored post-implantation training and rehabilitation programs are pivotal in optimizing outcomes, and facilitating patients’ adaptation to and proficient utilization of their CIs [10]. Additionally, the timing of implantation plays a pivotal role, with younger recipients typically exhibiting more favorable outcomes compared to older counterparts [11]. However, inherent patient variability necessitates a nuanced approach, recognizing the individual’s natural ability to tolerate and harness CI technology, thus underlining the importance of comprehensive assessment in CI candidacy determination and intervention planning.

**Figure 1:**
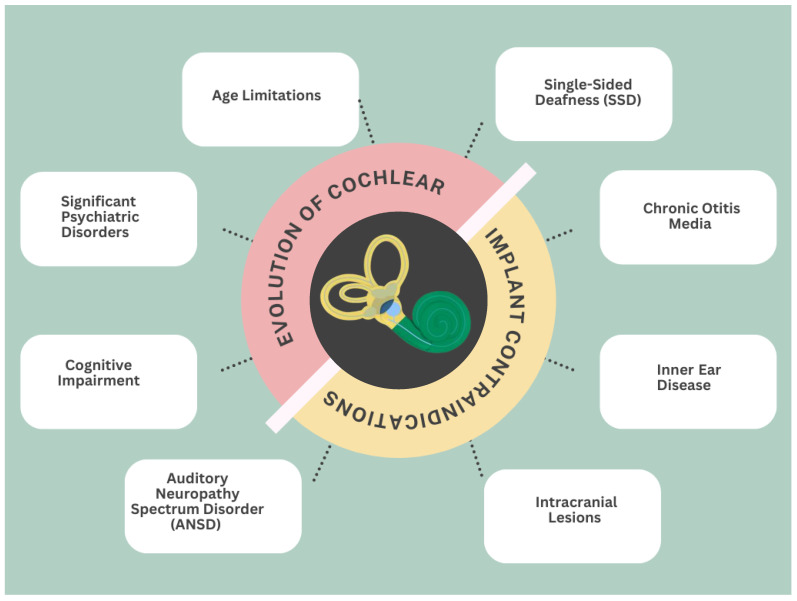
A concise overview of cochlear implant contraindications.

**Figure 2:**
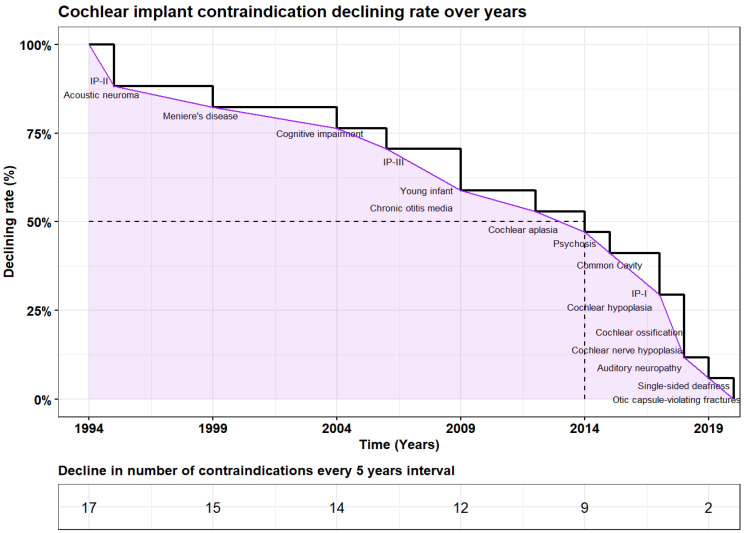
The figure illustrates the decline rate (%) in the number of cochlear implant contraindications over 25 years from 1994 to 2019. Each cycle represents a reduction in some contraindications mentioned on the decline curve, starting from 1994, until a complete resolution of all cochlear implant contraindications addressed in the literature by 2019. Additionally, the figure displays the median decline rate, with a 95% confidence interval (15–24 years), after 20 years of investigation, specifically in 2014. This is represented by the vertical dashed line intersecting the *x*-axis and the decline curve, adjacent to the 50% point on the *y*-axis.

This narrative lays the foundation for a comprehensive review that delves into the transformative journey of CI, emphasizing paradigm shifts in contraindications driven by advancements in technology, surgical techniques, and audiological considerations (Figure 1 and Figure 2).

## 2. Cochlear Implant Contraindications

### 2.1. Age Limitations

Over the years, CI candidacy criteria have undergone significant expansions, reflecting advances in pediatric hearing rehabilitation. The eligibility criteria, as outlined by FDA guidelines, have undergone significant expansion. Before 1985, CI was not offered to children, but eligibility gradually extended to those aged 2 years and older by 1990. A crucial development occurred in 2020 when the eligibility age was further reduced to 9 months [12]. Additionally, a previous study delved into the influence of CI timing on the development of the central auditory system, utilizing P1 cortical auditory evoked potential latency as a marker of maturity. Their findings highlighted a crucial period of around 3.5 years, during which the central auditory system exhibited maximum plasticity in the absence of standard stimulation. Moreover, plasticity persisted in some children until approximately age 7, beyond which it substantially diminished. These insights carry noteworthy implications for determining the most suitable age for CI in congenitally deaf children [13]. Furthermore, feasibility studies demonstrated the success of CI in infants under 6 months, with favorable outcomes and no increased complications [14,15]. Notably, infants as young as 4 months have received CI, reflecting a growing acceptance of early intervention [15]. The rationale for initiating CI at such early ages is grounded in the recognition that the brain’s heightened capacity for language acquisition during infancy, coupled with optimal neuronal priming, contributes to more favorable outcomes [16]. The shift toward earlier implantation aligns with the perspective that intervening at younger ages not only facilitates language development but also harnesses the neuroplasticity of the developing brain, crucial for successful language learning during this critical period [16,17]. Early CI programs emphasize the pivotal role of initiating access to CIs before the age of 9 months in fostering optimal language development in children [18].

In the realm of treating adolescents with prelingual hearing loss, the duration of auditory deprivation significantly influences the potential for post-surgery speech recognition. The decision to recommend CIs for a diverse group demands meticulous consideration, taking into account factors such as etiology, duration of hearing impairment, cognition, language, and the intricate interplay of parental expectations, family dynamics, the patient’s aspirations, social relationships, and identity implications. Historically, CIs were regarded as inappropriate for adolescents with prelingual hearing loss, a contentious practice confined to specific public hospitals [19]. Nevertheless, advancements in speech processors have reshaped this perspective, permitting a degree of speech recognition in this demographic.

Historically, perspectives on CI in the elderly have undergone a transformation from initial hesitancy to cautious optimism, largely influenced by a deeper understanding of age-related alterations in the auditory pathway. Research findings have provided insights into the effects of aging on both the peripheral auditory system, marked by a decrease in spiral ganglion cell counts in the cochlea and the central auditory pathways. The assessment of CI’s safety in the elderly indicates a favorable tolerance with comparable complication rates to younger adults, emphasizing the overall low risk, including that associated with general anesthesia. Advances in surgical techniques, coupled with the use of conscious sedation and local anesthesia, contribute to positive outcomes and minimal anesthesia-related risks [20,21]. While speech perception outcomes generally exhibit significant improvement, challenges persist in complex listening situations. Long-term studies validate sustained benefits in speech perception for up to five years post-implantation, with consistent reports of an enhanced quality of life from elderly CI recipients. The evolving landscape of CI in the elderly emphasizes its potential to address age-related hearing loss and associated challenges [22,23]. Furthermore, evidence shows that prelingually deafened adult patients can experience significant improvement through CI [24,25]. The decision to recommend CI for prelingually deafened adults requires careful consideration. In essence, CI surgery may be advised for prelingually deafened adults who have undergone effective habilitation, including consistent auditory verbal/oral training with well-fitted hearing aids [24]. In addition, previous research highlights a significant enhancement in both quality of life and speech recognition among prelingually deafened adults following CI surgery [25].

The aforementioned shift in perspective necessitates a reevaluation of the criteria for success and benefits, prompting a restructuring of pre-implant evaluations. The focus extends beyond mere auditory enhancement, emphasizing language assessment, social integration, and expectations regarding CI utilization, even for those already employing hearing aids. The primary objective of CIs in adolescents with prelingual hearing loss is to enable the perception and recognition of speech. A prior study revealed that the post-implant hearing thresholds of adolescents were adequate for accessing speech sounds. Despite variable outcomes, statistical improvements were evident across all employed speech tests, signifying a favorable impact of CIs on speech recognition in this population [19].

### 2.2. Single-Sided Deafness (SSD)

Individuals with unilateral deafness and contralateral-normal to near-normal hearing were initially excluded as candidates for CIs due to doubts about the perceived limited benefits of CIs and the belief that the presence of one functional ear would provide sufficient hearing ability. However, recent studies have shown the potential benefits of CIs in improving sound localization and speech understanding in individuals with SSD, particularly in noisy environments [26]. The classification of SSD as a contraindication varied among CI centers in the early 2000s, but a gradual shift in perspective occurred over time. The pivotal FDA approval of CIs for SSD in 2019 further endorsed its efficacy [27], solidifying the expanded scope of CI surgery for individuals with SSD and resulting in favorable outcomes in terms of auditory improvement and patient satisfaction. In the comparison between CIs and bone conduction devices (BCDs), the discrepancies became most apparent when listening in noisy environments. A meta-analysis revealed that CIs provide substantial advantages in terms of sound localization, tinnitus suppression, and overall quality of life assessment, while BCDs outperform in discerning speech in noisy environments and evaluate the quality of life related to background noise [28].

### 2.3. Chronic Otitis Media (COM)

Profound sensorineural hearing loss (SNHL) in COM patients, stemming from labyrinthitis, labyrinthine fistula, or iatrogenic injury, raises concerns during CI candidacy evaluations [29]. Historically, CI has been deemed contraindicated due to apprehensions about associated risks, encompassing cholesteatoma recurrence, meningitis, electrode extrusion, and suboptimal surgical outcomes [30]. However, recent decades have witnessed a paradigm shift, recognizing individuals with profound SNHL from COM as viable candidates for CI [31,32]. The management of these cases presents challenges, underscoring the imperative need to ensure the absence of ear infection before CI to minimize potential complications. The surgical approach to CI is meticulously tailored to each patient, taking into consideration the degree of COM activity. In instances of inactive COM with a dry perforation, a feasible option involves a single-stage procedure encompassing CI placement and closure of the tympanic membrane. Conversely, active infections necessitate staged procedures typically conducted post clearance of active COM. Aggressive management strategies, such as subtotal petrosectomy, involving mastoid sealing and protection of the electrode array, are implemented to ensure safety and efficacy [33,34]. Long-term follow-ups play a crucial role in assessing potential complications. Despite variations in surgical techniques, studies affirm the safety and feasibility of CI in COM, demonstrating hearing outcomes comparable to the general CI population [35].

In the realm of otitis media with effusion (OME) patients, CI emerges as a viable option, showing no significant rise in post-operative complications. Classifying patients by middle ear status (aerated, OME with grommet, untreated OME) revealed comparable complication rates during both early and late post-operative phases. Notably, the untreated OME group exhibited a higher incidence of intraoperative edema and granulation. Despite a 46% reduction in these issues among patients with ventilation tube insertion, the difference remained statistically insignificant compared to the untreated group. These results underscore the potential advantages of CI in OME patient care [36,37].

### 2.4. Inner Ear Diseases

#### 2.4.1. Trauma or Fracture of the Cochlea

The landscape of CI in patients with cochlear trauma has undergone a significant paradigm shift, challenging traditional beliefs. Despite otic capsule-violating fractures being historically deemed less favorable for successful CI, recent research has defied this notion, achieving success in 82% of cases with otic fractures, comparable to otic capsule-sparing fractures [38]. This transformative perspective underscores the evolving potential of CI for individuals with cochlear trauma. In the context of temporal bone fractures, several crucial considerations influence CI success. A prerequisite for successful implantation is an intact auditory nerve, which may be compromised by traction or avulsion injuries. Therefore, a meticulous pre-operative assessment involving imaging, audiology, and sometimes promontory stimulation becomes imperative to ensure the integrity and functionality of auditory nerves. Intra-operatively, factors like fracture lines, ossification, and fibrosis can distort anatomy, posing challenges for access and insertion. Consequently, a diverse range of approaches is often necessitated, with reported instances of incomplete electrode insertion highlighting the intricacies involved [39]. Moreover, in the case of profound hearing loss resulting from bilateral inner ear fractures, the consideration of CIs is warranted. A prior study has endorsed the adoption of bilateral CIs for individuals with fractures affecting both inner ears, emphasizing the importance of maximizing the benefits derived from CI surgery [40].

#### 2.4.2. Cochlear Ossification

Cochlear ossification (CO) refers to the abnormal growth of bone within the perilymphatic spaces of the cochlea as a result of inflammation or damage [41]. Initially, it was believed that the presence of ossification would impede electrode insertion and limit the effectiveness of CI. Despite the associated surgical challenges, CI in cases of CO has proven to be feasible usually. Successful creation of a patent cochlear lumen can be achieved through careful drilling in cases of ossification around the round window or basal turn, extending up to the ascending basal turn. In instances of complete scala tympani ossification, opting for insertion into the scala vestibule proves to be a viable alternative [42]. A previous study noted that although scala tympani insertion is considered the gold standard, scala vestibuli insertion does not adversely affect hearing outcomes, yielding comparable auditory performance in both quiet and noisy environments [43]. The degree of ossification in the basal turn appears to have minimal impact on auditory outcomes, as long as there is adequate electrode insertion into either scala. Notably, even one-year post-implantation, no significant differences were observed when compared to cases involving non-otosclerotic pathologies [42].

#### 2.4.3. Inner Ear Malformations (IEM)

The chronological evolution in CI contraindications associated with IEMs reflects a transformative journey. Originally, cases of congenital SNHL were broadly categorized into two groups. The majority of cases (80%) are associated with membranous malformations affecting the inner ear hair cells, without any significant abnormalities in the bony structure. The remaining 20% of cases encompass diverse malformations affecting the bony labyrinth, which can be visualized through radiological imaging techniques. Managing this group poses surgical challenges and decision-making complexities. Treatment options range from hearing aids to CI, and certain cases may be eligible for auditory brainstem implantation (ABI) [44].

Incomplete partition anomalies are a subset of cochlear malformations characterized by distinguishable cochlea and vestibule structures, which maintain normal outer dimensions but display abnormal internal architecture. A classification system has been established to categorize these incomplete partitions into three subgroups based on the specific defects involving the central modiolus and interscalar septa [44]. In type I, the cochlea resembles a cyst-like structure due to absence of the entire modiolus and interscalar septa. The presence of stapes footplate abnormality in this particular type increases the susceptibility to meningitis. In such cases, the cochlea may be completely filled with cerebrospinal fluid (CSF). To address this issue, FORM 24 (Med El) has been developed, featuring a conical stopper that effectively prevents CSF leakage around the electrodes. Similarly, Digisonic Classic and Digisonic Evo electrodes (Oticon) also incorporate a silicon stopper, which could be beneficial in managing cases of gusher [44]. Whereas type II IP malformation occurs when there is a defect in the apical region of the modiolus and the corresponding interscalar septa resulting in an abnormality where the apex of the cochlea appears cystic due to the merging of the middle and apical turns.

Previously, CI was thought to be contraindicated for cases with cochlear malformation [45]. Mangabeira-Albernaz holds the distinction of being the first to conduct CI procedures on patients with cochlear malformations, specifically those classified under the umbrella term “Mondini Dysplasia”. Subsequently, a considerable number of CIs have been documented, yielding favorable outcomes [45,46,47,48]. In cases where the basal part of the modiolus remains unaffected, a variety of electrodes can be used during CI. As some patients might experience severe gusher episodes during the surgical procedure, it is advisable to use an electrode equipped with a silicon stopper for type II IP cases [44]. On the other hand, type III IP occurs when the cochlea has interscalar septa and no modiolus. In the past, CI was contraindicated to be performed in patients with type III IP due to the high risk of CSF gusher during CI surgery. Because the majority of patients in this group will have CSF gusher during CI and a high chance of electrode misplacement in the IAC, FORM 24 electrodes are advised to effectively address both concerns [44,45,46,47,48,49].

Michel deformity manifests as a total absence of both cochlear and vestibular structures [45]. The visualization of the facial canal in the temporal bone may prove challenging, notwithstanding normal facial functions. Moreover, audiological evaluations may not yield a response or may indicate profound SNHL [44]. It has been found that it is not possible to perform CI on patients with Michel deformity and undergoing ABI is the best management option [44].

Cochlear aplasia is a complete absence of the cochlea. Children with cochlear aplasia comprised 2% of cases with inner ear malformations and constituted 0.4% of the entire pediatric CI recipient population [50]. Patients with cochlear aplasia can be categorized into cochlear aplasia with normal labyrinth and cochlear aplasia with a dilated vestibule. These patients previously were primarily managed by ABI and not CI as it was deemed contraindicated [44,50]. Recent studies indicate that children with cochlear aplasia exhibited preoperative audiological responses, confirming the eighth nerve’s presence in imaging. Following implantation, neural responses were evident, improving hearing thresholds to 25 dB HL, enabling significant open-set speech perception [50]. Moreover, a case report outlines the experience of a child with bilateral cochlear aplasia who underwent unilateral CI surgery. The child demonstrated favorable audiological and speech progressions, achieving a 200-word vocabulary and the capacity to form two-word sentences at the one-year follow-up [51]. Previous research has indicated that CI surgery yields audiological and speech advantages for individuals with cochlear aplasia; however, these benefits are noted to be less favorable compared to CI surgery outcomes in individuals with normal inner ears [51,52]. Despite challenges in subgrouping cystic cavities in inner ear malformation cases, the report emphasizes that such complexities should not deter the consideration of CI surgery, especially when there is an acceptable anatomical appearance, the presence of the cochleovestibular nerve, and the use of an appropriate electrode [51].

The common cavity (CC) deformity is defined as a cystic cavity representing the coalescence of cochlea and vestibule; the internal auditory canal (IAC) undergoes normal development and the cavity is typically situated at its central position [44]. Previously, it was difficult to locate the cochlear ganglion and CI was contraindicated due to facial nerve injury risk and CSF gusher [45,53]. Recent studies have underscored the effectiveness of CI as a therapeutic measure for children with CC. Although their audiological development may not attain the level observed in those without CC, a significant number of subjects derive notable benefits from this intervention [54,55]. CIs notably enhance awareness of environmental sounds and contribute to the development of a limited vocabulary [56]. While patients with CC deformity exhibited beneficial audiological and speech outcomes, performance variability was notable, emphasizing the need for pre-operative counseling for parents. The choice of surgical approach should be tailored based on individual clinical, radiological, and surgical findings [55].

Cochlear hypoplasia is marked by the differentiation of malformation to the extent that the cochlea and vestibule become distinct entities but with dimensions smaller than the norm. Originally, CI was deemed contraindicated in these cases given reports of increased risk of electrode insertion into the internal auditory canal due to small cochlear dimensions and facial nerve anomalies [47]. However, current approaches include utilizing hearing aids and stapedotomy for conductive components, with CI now considered a viable alternative for profound SNHL. Patients with deficient cochlear nerves may benefit from ABI. When CI is pursued, it is recommended to employ short, thin electrode arrays to avoid CSF gusher [44]. Careful patient selection through multidisciplinary evaluation can optimize outcomes. A recent study introduces a novel classification of cochlear hypoplasia. It recommended determining the length of the electrode array by evaluating the observed length of the cochlear lumen in 3D imaging [57].

#### 2.4.4. Ménière’s Disease (MD)

MD is an idiopathic inner ear condition characterized by spontaneous episodic attacks of vertigo, sensorineural hearing loss, ear fullness, and tinnitus [58]. Potential etiologies include endolymphatic hydrops due to ischemic injury, or autoimmune processes within the inner ear [59]. Studies showed that individuals with unilateral MD and profound SNHL, who underwent CI surgery either with or without concurrent labyrinthectomy, demonstrate effective management of their condition. This approach has demonstrated enhancements in speech comprehension, vertigo control, tinnitus relief, and overall quality of life when utilizing the device [60,61]. Therefore, CI extends similar functional benefits for auditory rehabilitation in individuals with MD as it does for those with other causes of hearing loss [60,61].

### 2.5. Cochlear Nerve Hypoplasia and Aplasia

Congenital profound deafness occurs in around 2% of cases due to deficits of the cochlear nerve [62]. Although imaging is crucial for evaluating the nerve, especially in cases with a narrow internal auditory canal (IAC), its resolution lacks the precision needed to distinguish between hypoplasia and aplasia. Consequently, a comprehensive audiological evaluation, incorporating electrically evoked auditory brainstem response testing conducted through either transtympanic stimulation at the round window or with an intracochlear test electrode, is warranted even when imaging implies cochlear nerve aplasia [62]. This is because auditory function may still exist. If the results of any test suggest the cochlear nerve is present, CI can still be offered. In a recent investigation, it was observed that following CI, approximately 50% of individuals with cochlear nerve aplasia and 90% of those with CN hypoplasia achieved some degree of speech understanding [63].

### 2.6. Auditory Neuropathy Spectrum Disorder and Limited Auditory Nerve Function

Auditory neuropathy spectrum disorder (ANSD) is characterized by abnormal auditory nerve function despite intact cochlear hair cell function. Individuals with severely compromised or absent auditory nerve function were initially considered contraindications, due to concerns about the feasibility of electrical stimulation in these cases, as the CI relies on the stimulation of the auditory nerve to transmit sound signals to the brain. Thus, advances in CI technology, such as ABIs, have expanded options for individuals with limited auditory nerve function [44]. ANSD is typically diagnosed around 11 months, distinguished by the presence of OAEs alongside abnormal ABRs. Audiological evaluations play a pivotal role in both diagnosing ANSD and determining suitable treatment pathways, including options such as hearing aids, CIs, or ABIs. Furthermore, the consideration of additional tests like eABR and cortical potentials is essential for optimizing patient management strategies. The audiological assessment encompasses various diagnostic modalities, including stapedial reflex measurements, supraliminal psychoacoustic tests, electrocochleography, auditory steady-state responses, and cortical auditory evoked potentials [64]. While traditional hearing aids are frequently recommended for ANSD, empirical evidence consistently highlights their limited efficacy. Conversely, recent investigations revealed that CIs are a viable therapeutic option for individuals with ANSD. However, CI outcomes may be influenced by cochlear nerve aplasia or hypoplasia [65,66]. CI has been shown to provide similar benefits in children with ANSD as in children with profound SNHL [67,68,69]. Crucially, it is imperative to distinguish ANSD from conditions such as auditory processing disorder (APD) and hidden hearing loss (HHL). APD and HHL predominantly influence speech intelligibility in challenging circumstances rather than pure tone thresholds. While certain cases initially diagnosed as ANSD may subsequently align more closely with APD criteria, clinicians aim to differentiate between these conditions and precisely identify auditory deficits within the nervous system [70].

### 2.7. Intracranial Lesions

The landscape of CI surgery post-tumor treatment has undergone a revolutionary shift in hearing rehabilitation for neurofibromatosis type 2 (NF2) and vestibular schwannoma (VS) patients. While ABIs were conventionally favored, recent progress establishes CIs as a robust alternative, delivering a spectrum of auditory benefits from heightened sound awareness to impressive telephone use outcomes [71]. Preoperative audiological assessments, such as eABR and promontorium stimulation tests, along with imaging studies, can effectively demonstrate the anticipated benefits of CI in the postoperative phase. Challenges in this regard encompass surgical intricacies, radiation therapy impacts, and the secure application of MRI for CI recipients. CIs rely on an intact or near-intact cochlear nerve, posing challenges for CI candidacy in only a minority of NF2 cases due to difficulties in nerve preservation. Despite bilateral intracranial tumors, CIs typically yield better auditory outcomes than ABIs. However, tumor progression can adversely affect CI function over time [72]. A preceding investigation revealed that the translabyrinthine surgical approach does not negatively affect speech test outcomes [71]. Furthermore, radiosurgery demonstrates positive auditory outcomes [71,73]. On the other hand, among individuals with intracochlear schwannomas, there appears to be a notable trend toward improved word recognition performance with CIs, even in cases involving significant structural damage to the cochlear capsule due to partial or subtotal cochleoectomy [74]. The unexpectedly positive functional outcomes, despite considerable trauma to the cochlea, may challenge prevailing clinical perspectives on CI surgery, extending beyond the scope of this specific indication [74].

The application of MRI in conjunction with CIs may lead to the generation of artifacts, posing a risk of obscuring essential pathological findings, particularly in patients with neurological conditions like NF2 or infratemporal meningiomas, despite the compatibility of CIs with MRI. Noteworthy, previous study underscores the importance of considering surgical removal of the CI when compromised MRI images necessitate precise diagnosis and management, especially in patients at risk of multiple intracranial lesions like NF2 [75]. Postoperative care and the assessment of intracerebral structures post-CI surgery are increasingly crucial with the expanding global indications for CIs. The strategic placement of a magnet-induced artifact, at least 9 cm from the outer ear canal, enables the examination of the labyrinth and internal auditory canal [76]. In addition, undergoing an MRI can cause significant discomfort for individuals with CIs; nevertheless, MRI scans up to 1.5 Teslas, conducted with external head fixation, are considered safe for CI recipients [71].

### 2.8. Significant Psychiatric Disorders

Individuals with severe psychiatric disorders, such as schizophrenia or severe depression, were initially excluded as candidates for CI due to concerns about their ability to comply with the post-implantation rehabilitation process and effectively utilize the device. According to a recent study in adults, significant alleviation of depressive symptoms was observed at the 6-month mark following treatment in individuals who underwent CI and used hearing aids [77]. A recent meta-analysis discovered a noteworthy, heightened risk of psychosis, delirium, and schizophrenia among individuals with hearing loss. Loneliness and social isolation are potential mechanisms that may underlie this association [78]. A case study examined three patients with multiple and diverse psychiatric disorders who underwent CI. All three patients opted for a second implant and achieved positive hearing outcomes. None of the patients encountered acceptance issues, and there were no long-term exacerbations of their psychiatric conditions [79].

### 2.9. Cognitive Impairment

Individuals with significant cognitive impairments were initially disqualified from CI due to concerns about their ability to adapt to the device and participate in rehabilitation. A previous study indicated that individuals with mild to moderate mental retardation experience significant advancements in speech perception during the initial two years after CI [80]. Additionally, another study revealed that children with mental retardation derive measurable benefits from CIs, with their postoperative performance influenced by the severity of their condition [81]. In addition, basic intellectual skills can significantly impact the outcomes of CI users. Research has shown that cognitive abilities, including language comprehension and processing speed, play a crucial role in the success of CI interventions [82]. Therefore, it is essential for clinicians to consider the cognitive profile of CI candidates during pre-implantation assessment and post-implantation rehabilitation to optimize outcomes and support the individual’s auditory development. The objectives of cochlear implantation in infants with profound SNHL and concurrent central nervous system disorders are diverse and contingent upon various factors, including the severity and nature of the neurological condition. While the primary aim remains to facilitate auditory development and enhance communication skills through sound access, additional goals may involve addressing cognitive or developmental challenges associated with the central nervous system disorder.

Organic brain syndrome is a term that covers various disorders with different types of mental symptoms (such as confusion, memory loss, delusions, hallucinations, mood changes, and personality changes) [83]. One study aimed to assess the effect of CI, as a form of hearing rehabilitation, on cognitive decline in older adults. Post implantation, significant improvements were observed in speech perception, quality of life, and neurocognitive abilities at the six-month mark. The most notable advancements were found in executive functions, particularly attention, inhibition, and working memory. Delayed recall also displayed a significant improvement. However, changes in long-term memory performance were only evident after 12 months [84].

## 3. Conclusions

This narrative review offers a comprehensive examination of the chronological contraindications linked to CI, emphasizing the evolving perspectives over time (Table 1, Figure 1 and Figure 2). Nevertheless, some conditions, notably untreated intracranial lesions, still stand as contraindications for CI. The reevaluation of contraindications has opened new possibilities and expanded indications for cochlear implantation. Emerging prospects and advantages, such as improved outcomes and enhanced quality of life, offer hope for individuals with profound SNHL. As the field continues to advance, collaborative efforts among clinicians, researchers, and policymakers are crucial to further refine and optimize the decision-making process in cochlear implantation. Ensuring adherence to stringent criteria for CI eligibility is imperative to mitigate complications and optimize outcomes. While CI devices may not entirely restore normal hearing, they serve as pivotal tools in auditory rehabilitation, significantly augmenting recipients’ hearing capabilities and overall quality of life.

## Figures and Tables

**Table 1:** Summary of the historical complications of cochlear implantation with rationale behind it and reasons to remove it.

Contraindication		Rationale Behind It	Rationale to Eliminate It
Age limitation	Young age	Risks of anesthesia and surgery and limited understanding of auditory physiology.	Advancements in anesthesia and surgical expertise have resulted in safer outcomes. Additionally, younger children, particularly those aged 6–9 months, have shown positive auditory outcomes.
	Adolescents	Thoughts on limited neural plasticity and reduced capacity to develop linguistic skills compared to younger patients.	The development of speech processors enhances speech recognition capabilities.
	Elderly	Risk of anesthesia and surgery and the belief of poor outcomes due to the effects of aging on the peripheral auditory system.	Advances in surgical and anesthesia techniques have resulted in minimal risks. Additionally, long-term studies have shown positive outcomes in speech perception and overall quality of life.
Single-sided deafness		Uncertainty regarding the unilateral perceived.	CIs have shown improvements in sound localization and tinnitus suppression.
Chronic otitis media		Risks of cholesteatoma, recurrence, meningitis, electrode extrusion, and suboptimal outcomes	Advancement in surgical expertise has led to various approaches for treating COM, including one-stage, two-stage, or subtotal petrosectomy, based on the extent of the condition.
Inner ear diseases	Trauma or fracture of the cochlea	An otic violation fracture can cause permanent damage to the sensory organs.	Direct nerve stimulation has demonstrated a high success rate. Meticulous pre and intra-operative factors are essential for improved outcomes.
	Cochlear ossification	Beliefs regarding the inability to insert the electrode into the ossified lumen.	New techniques, such as the creation of cochlear lumen or insertion into scala vestibuli, have emerged, resulting in a high success rate.
	Inner ear malformations	Limited data on the benefits, as well as the risks of CSF gusher, meningitis, facial nerve injury, and false insertion.	Careful patient selection, verification of vestibulocochlear nerve presence, proper planning for electrode type, consideration of a gusher stopper if needed, and implementation of precautionary measures.
	Ménière’s disease	Misleading notion of insufficient spiral ganglion cells due to disease progression or after labyrinthectomy.	Data indicate significant improvements in speech comprehension, vertigo control, tinnitus relief, and overall quality of life.
Cochlear nerve hypoplasia and aplasia		A belief that nerve activity is completely impaired or malfunctional.	Some residual auditory function may present, necessitating comprehensive testing through methods such as round window or intracochlear testing.
Auditor neuropathy spectrum		The perception of limited effectiveness of electrical stimulation.	The outcomes are influenced by the integrity of the nerve, which can result in positive outcomes.
Intracranial lesions		Limited exposure to radiotherapy and challenges in surveillance due to MRI artifacts.	Translabyrinthine surgery and radiation have shown positive auditory outcomes. CIs are also compatible with MRI scans. However, it is important to assess the need for surveillance MRI prior to undergoing CI.
Significant psychiatric disorders		There are concerns regarding inadequate adherence to post-implant rehabilitation protocols.	Studies showed positive outcomes in depression and psychosis.
Cognitive impairment			A positive outcome has been observed in individuals with mild to moderate cognitive impairment. In cases of severe cognitive impairment, cochlear implants can offer benefits such as environmental awareness and safety.

## Data Availability

No new data were created or analyzed in this study. Data sharing is not applicable to this article.
